# A prospective study on inter-operator variability in semi-robotic software-based MRI/TRUS-fusion targeted prostate biopsies

**DOI:** 10.1007/s00345-021-03891-3

**Published:** 2021-11-26

**Authors:** Fabian Derigs, Samuel Doryumu, Fabian Tollens, Dominik Nörenberg, Manuel Neuberger, Jost von Hardenberg, Maurice Stephan Michel, Manuel Ritter, Niklas Westhoff

**Affiliations:** 1 Department of Urology and Urosurgery, Medical Faculty Mannheim, Heidelberg University, Mannheim, Germany; 2 Department of Radiology and Nuclear Medicine, University Medical Center Mannheim, Medical Faculty Mannheim, Heidelberg University, Mannheim, Germany; 3 Department of Urology and Pediatric Urology, University Hospital of Bonn, Bonn, Germany; 4 Department of Urology and Urosurgery, University Medical Center Mannheim, Theodor-Kutzer-Ufer 1-3, 68167 Mannheim, Germany

**Keywords:** Prostatic neoplasm, Multiparametric magnetic resonance imaging, Software-based fusion biopsy, Reproducibility, Surgeon, Accuracy

## Abstract

**Purpose:**

Magnetic resonance imaging **(**MRI)/ultrasound-fusion prostate biopsy (FB) comprises multiple steps each of which can cause alterations in targeted biopsy (TB) accuracy leading to false-negative results. The aim was to assess the inter-operator variability of software-based fusion TB by targeting the same MRI-lesions by different urologists.

**Methods:**

In this prospective study, 142 patients eligible for analysis underwent software-based FB. TB of all lesions (*n* = 172) were carried out by two different urologists per patient (*n* = 31 urologists). We analyzed the number of mismatches [overall prostate cancer (PCa), clinically significant PCa (csPCa) and non-significant PCa (nsPCa)] between both performed TB per patient. In addition we evaluated factors contributing to inter-operator variability by uni- and multivariable analyses.

**Results:**

In 11.6% of all MRI-lesions (10.6% of all patients) there was a mismatch between TB1 and TB2 in terms of overall prostate cancer (PCa detection. Regarding csPCa, patient-based mismatch occurred in 14.8% (*n* = 21). Overall PCa and csPCa detection rate of TB1 and TB2 did not differ significantly on a per-patient and per-lesion level.

Analyses revealed a smaller lesion size as predictive for mismatches (OR 9.19, 95% CI 2.02–41.83, *p* < 0.001).

**Conclusion:**

Reproducibility and precision of targeting particularly small lesions is still limited although using software-based FB. Further improvements in image-fusion, segmentation, needle-guidance, and automatization are necessary.

**Supplementary Information:**

The online version contains supplementary material available at 10.1007/s00345-021-03891-3.

## Introduction

Multiparametric magnetic resonance imaging (mpMRI) in combination with targeted biopsy (TB) has greatly improved the identification of clinically significant prostate cancer (csPCa) [1]. Thus, MRI/ultrasound-fusion biopsy (FB) has been widely introduced in the last decade. Software-based image-fusion has gained greatest acceptance of fusion techniques [2].

Although TB has increased the detection rate for csPCa, yet a considerable amount of csPCa still remains undetected by TB [3–5]. This innovative approach of biopsy sampling represents a multi-step procedure involving different disciplines. Each step requires its own expertise which implies the occurrence of variations in the process. One of the preconditions for optimization of TB is the identification of its weaknesses to ensure no missing of csPCa.

The extent of inter-reader variability between radiologists and its implications for detection of prostate cancer (PCa) has already been described [6]. MRI result reporting from radiologists has also been shown to be of importance for biopsy performance [7]. Losses in accuracy in TB sampling have been analyzed in former research showing that experience of the urologist might also play an important role in cancer detection rates, CDRs [8, 9]. However, when evaluating inter-operator variability in FB, most studies compared CDR of urologists with different levels of experience on different patients [8–10].

The aim of this prospective study was to assess the inter-operator variability and reproducibility of software-based fusion TB by targeting the same lesions by different urologists.

## Methods

### Study design

This prospective study was approved by the Local Institutional Ethical Review Board (approval no. 2015-403M-MA). All patients signed written informed consent for the intervention. Recruitment occurred at the University Medical Center Mannheim (Germany) between October 2016 and March 2021.

### Study population

All men (≥ 18 years of age) with (i) PCa suspicion [abnormal digital rectal examination, prostate specific antigen (PSA) elevation or abnormal MRI], (ii) persistent suspicion after one or more negative prior biopsies, or (iii) control biopsy while undergoing active surveillance, were eligible for inclusion.

### Acquisition and reporting of mpMRI

A mpMRI was performed in all patients either in the in-house radiology department (*n* = 70) or external facilities (*n* = 65). For mpMRI acquisition, a magnetic field-strength of 3.0 T (Magnetom Skyra and Trio, Siemens Healthineers, Erlangen, Germany) was used at the in-house department and either 1.5 T or 3.0 T at external departments, mostly without use of an endorectal coil. T2-weighted sequences, diffusion-weighted imaging (DWI; b-values of 50, 400, 800 s/mm^2^, additional *b*-value of 2000s/mm^2^ for Magnetom Skyra), and dynamic contrast enhanced perfusion sequences were obtained. Images were read and interpreted by respective uroradiologists who performed the mpMRI. In-house radiological appraisal was carried out or supervised by uroradiologists with more than 5 years of experience in urogenital imaging. MRI-lesions were scored according to latest PI-RADS guidelines (version dependent on the time of image acquisition).

### MRI/TRUS-fusion biopsy

FB was performed under local or general anesthesia using the software-based robotic-assisted Artemis™ platform (Eigen, USA). Patients received either prophylactic or targeted antibiotic treatment dependent on preoperative rectal swap (and urine culture in case of risk factors for urinary tract infections). TB was performed independently by two urologists (*n* = 31 urologists) per patient. The first urologist contoured the prostate as well as suspicious lesion(s) within the MR images using the respective fusion software Profuse™. Contouring was performed on the T2-weighted sequence as requested by taking also diffusion-weighted image and dynamic contrast-enhanced sequences into account. Afterwards, this urologist created a 3D-model of the prostate by the TRUS scan. After performing the TB of all lesions (TB1), the first urologist left the operating room, and the second urologist re-started the biopsy session with a new TRUS-scan and image-fusion procedure. TB sampling of the same lesions (TB2) was followed by the 12-core SB done by the second urologist.

### Data analysis

Demographic, clinical, imaging and histopathological data were assessed by descriptive analysis. CDRs were analyzed on per-patient and per-lesion levels. An ISUP ≥ 2 PCa was defined as clinically significant. Primary outcome was the number of mismatches [overall PCa, csPCa and non-significant PCa (nsPCa)] between TBs of both urologists per patient. Secondary outcomes were factors that contribute to inter-operator variability in TB.

CDR were compared between biopsies using McNemar test. Cohen’s *κ* statistic was used for calculation of inter-operator variability between the two urologists. Potential predictors for the occurrence of discrepancy between biopsy results were calculated by univariable analyses. Variables showing an odds ratio of > 1.5 were further tested by multivariable analyses. For these calculations all lesions of PCa negative patients were excluded. For comparison of qualitative parameters Fisher’s exact test was used. Experience as a factor for potential mismatches was evaluated by assessing the difference of the individual number of previously made in-house FB.

Analyses were performed using JMP® 15.0.0 and IBM® SPSS® Statistic Version 27 software. Level of statistical significance was set at *p* < 0.05.

## Results

Characteristics of the study population are shown in Table 1. Overall, 155 patients received an MRI/TRUS-fusion biopsy and signed the informed consent for the study. Patients who either had no complete study biopsy by a second urologist (*n* = 11) or received a control-biopsy after focal therapy (*n* = 2) were excluded.

**Table 1:** Patient characteristics, results of mpMRI and MRI/TRUS fusion biopsy

Variable	*n* = 142 Patients
Patient characteristics	
Age, year (median, IQR)	68.5 (61–74)
PSA, ng/ml (median, IQR)	7.3 (5.4–10.6)
Prostate volume, cm^3^ (median, IQR)	45.7 (35.0–64.7)
PSA-density, ng/ml^2^ (median, IQR)	0.16 (0.09–0.23)
Suspicious digital rectal examination (%)	29 (20.9)
Biopsy naïve (*n*, %)	91 (64.1)
Previous negative biopsy (*n*, %)	44 (31.0)
Previous positive biopsy (*n*, %)	7 (4.9)
Magnetic resonance imaging results	
Unifocal lesion (*n*, %)	115 (81.0)
Multifocal lesions (*n*, %)	27 (19.0)
Index lesions (*n*, %)	142
PI-RADS 5	40 (28.2)
PI-RADS 4	74 (52.1)
PI-RADS 3	27 (19.0)
PI-RADS 2	1 (0.7)
Total no. of lesions (*n*, %)	172
PI-RADS 5	41 (23.8)
PI-RADS 4	86 (50.0)
PI-RADS 3	43 (25.0)
PI-RADS 2	2 (1.2)
Max. diameter of index lesions, mm (median, IQR)	11 (7.5–15.0)
Biopsy results	
SB cores per patient (median, IQR)	12 (12–12)
Lesions with (*n*, %)	
2 TB cores per operator	136 (79.1)
3 TB cores per operator	31 (18.0)
4 TB cores per operator	5 (2.9)
TB positive per patient (*n*, %)	88 (62)
SB positive per patient (*n*, %)	92 (64.8)
TB positive per lesion (*n*, %)	96 (55.8)
SB cores positive for PCa/total SB cores (*n*, %)	284/1704 (16.67)
TB cores positive for PCa/total TB cores (*n*, %)	286/688 (41.69)
SB cancer core infiltration, mm (median, IQR)	5.8 (2.0–10.9)
TB cancer core infiltration, mm (median, IQR)	7.9 (4.2–11.2)
ISUP per patient^a^ (*n*, %)	142
I	28 (19.7)
II	45 (31.7)
III	14 (9.9)
IV	9 (6.3)
V	6 (4.2)
No cancer	40 (28.2)
ISUP per lesion^b^ (*n*, %)	172
I	31 (18.0)
II	32 (18.6)
III	8 (4.7)
IV	19 (11.0)
VI	6 (3.5)
No cancer	76 (44.2)
Only TB positive of (*n*, % of PCa positive patients)	10 (9.8)
Only SB positive (*n*, % of PCa positive patients)	14 (13.7)
Discrepancies in detecting PCa by TB (*n*, % from all PCa positive patients)	15 (14.7)

*PCa* prostate cancer, *PI-RADS* Prostate Imaging Reporting and Data System, *TB* targeted biopsy, *SB* systematic biopsy

^a^By systematic biopsy and targeted biopsy

^b^By targeted biopsy

The overall CDR was 71.8% (102/142 patients). SB found 64.8% PCa compared to 62.0% found by both TB (TB1 + TB2) (*p* = 0.541). Total csPCa detection rate (csCDR) was 52.1% (41.5% SB vs. 43.0% TB, *p* = 0.851). Out of 102 cancer positive biopsies on a per-patient level, 11 PCa (10.8%) were detected exclusively by TB (TB1 + TB2) and 14 PCa only by SB (13.7%) (*p* = 0.690). Out of 74 patients with a csPCa (SB + TB), 13 patients (17.6%) had a csPCa finding solely in SB cores and 15 (20.3%) only in TB cores (TB1 + TB2) (*p* = 0.851). On a per-lesion level, TB detection rates were 55.8% (CDR) and 37.8% for csPCa.

There was no significant difference in CDR between TB1 and TB2 in all subgroups. The comparison of patient- and lesion-based TB1 and TB2 detection rates is shown in Online Resource 1. The lesion-based degree of agreement in detecting overall PCa (*κ* = 0.56) and in detecting nsPCa (*κ* = 0.56) between TB1 and TB2 was by definition “moderate”. Agreement in csPCa detection was by definition “substantial” (*κ* = 0.65) (Online Resource 2).

**Fig. 1:**
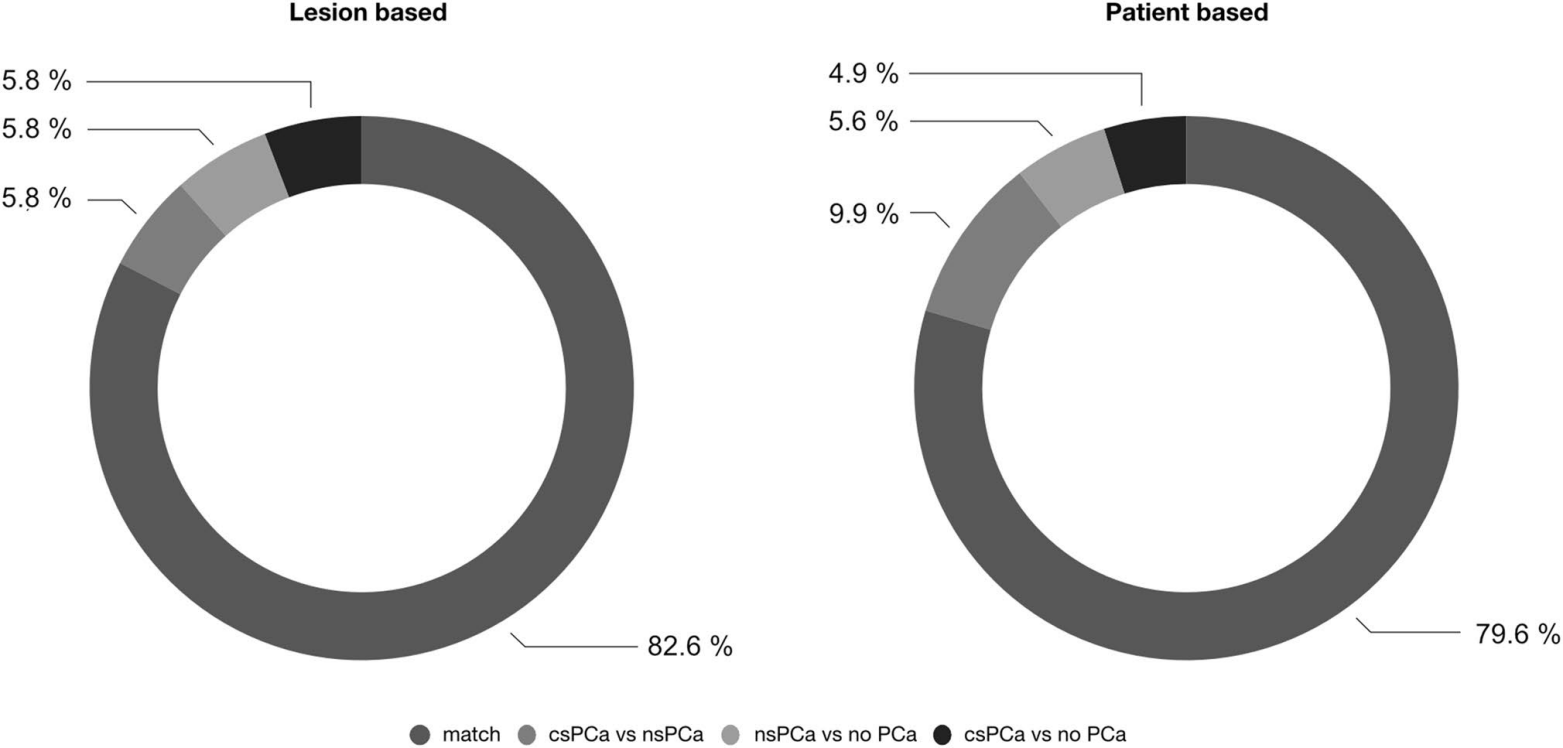
Number and types of mismatches (discrepancies in histopathological biopsy results) between targeted biopsy 1 (TB1) and targeted biopsy 2 (TB2)

Figure 1 illustrates the number and types of mismatches between TB1 and TB2. In 20 MRI-suspicious lesions (11.6%), corresponding to 15 patients (10.6%), there was a mismatch between TB1 and TB2 in terms of overall cancer detection. Two out of 15 patients, whose lesions were only hit by one TB (TB1 or TB2), had a negative SB and no other positive lesions. In terms of csPCa detection, there was a patient-based mismatch of 14.8% (*n* = 21) between TB1 and TB2. Six out of those 21 mismatch patients (4.2% of total patients) had a benign or clinically insignificant finding (ISUP = 1) in the SB.

Of all PCa positive lesions, in which both, TB1 and TB2, were cancer positive (*n* = 76), upgrading to a csPCa, either in TB1 or TB2, occurred in 13.2% (*n* = 10).

The univariable and multivariable analyses of 112 lesions from the PCa positive patients for factors associated with mismatches revealed the size of the lesion (≤ 12 mm) described in the MRI as a predictive variable for mismatches (*p* < 0.001). Higher prostate volumes (> 41.21 ml) (OR 2.15, 95% CI 0.79–5.84, *p* = 0.184) did not significantly correlate with mismatches (Online Resource 3).

Analyses of the 102 PCa positive patients revealed that the size of the lesion was significantly smaller (*p* = 0.005) and prostate volume was significantly bigger (*p* = 0.014) in the group of patients who were only cancer positive in SB (*n* = 14).

Adverse events occurred in 7.0% (*n* = 10) of patients. Hematuria was detected in 2.0% (*n* = 3), urinary retention in 2.0% (*n* = 3), rectal bleeding in 1.4% (*n* = 2) and fever in 1.4% (*n* = 2).

## Discussion

Despite the superiority of FB compared to SB, many high-risk PCa still remains undetected by TB as shown by Ahdoot et al. [3]. They showed a misclassification rate of up to 13.6% of all csPCa bearing patients if SB would have been omitted [3]. In our study cohort, 17.6% of PCa patients with ISUP-score ≥ 2 were misclassified by TB. Many factors have already been investigated which might influence FB [6–10]. These findings leave us the consideration of why the biopsy result of a suspicious lesion is negative.

The methodical approach of investigating inter-operator variability in the same patient by two different urologists to eliminate all confounding factors of procedure comparison has so far not been undertaken.

A key finding of our present study is that a considerable number of mismatches in PCa detection as well as in csPCa detection between both urologists could be observed. Although overall PCa and csPCa detection rate of TB1 and TB2 did not differ significantly on a per-patient and on a per-lesion level, a discrepancy in csPCa finding occurred in 14.8% of the study population. In total, a csPCa finding could have been missed in up to 4.2% of all patients, if the TB had been carried out only by one of the urologists. The remaining csPCa mismatch patients would have been covered by the SB, which accounts for 10.6%, underlining the importance SB still has in this setting. This result also suggests that more than two cores should be taken from each target lesion in FB. Even though the number of TB cores was not a predictor for occurrence of mismatches here, several trials showed that up to 10% of patients would benefit from more than two cores per lesion [11].

Compared to Ahdoot et al., we identified a similar yield of PCa (62.0% vs. 51.5%) and csPCa (43.0% vs. 37.8%) with TB in our study [3]. Although detection rates between both TB did not differ significantly, the lesion-based level of agreement was not optimal. In total, 20 lesion-based PCa detection discrepancies between TB1 and TB2 were found in our study. Half of those 20 lesions (*n* = 10) comprised a clinically significant cancer finding, emphasizing the imperfect reproducibility of TB even with the remarkable assistance of a biopsy platform.

In search of potential factors influencing the occurrence of mismatches between urologists, a smaller size of the MRI-lesion was revealed as a significant predictor (OR = 9.19). The size of the lesion is also associated with the likelihood of both urologists missing the target, which supports the assumption that smaller PCa lesions are less likely to be identified by TB. This finding is in agreement with the recent study of Baco et al. describing a reduced csPCa detection rate of 50% for lesions < 0.5 ml vs. 76% for lesions > 1 ml in size [12, 13]. It has been suggested that a perfect fusion of both images is necessary to reliably hit smaller targets. A small error in prostate and lesion boundary segmentation can already have a major impact on successful targeting [13]. The fact that both urologists in our study needed to carry out the delineation of the prostate in TRUS images, a procedure which requires high precision and is thus a source of targeting error, could explain these findings. The number of mismatches might be even higher if contouring of prostate and lesions in MRI-images was also done by each urologist separately. As discussed by Tay et al., ultrasound-segmentation of the prostate necessitates a smooth and even sweep by the probe to avoid any displacements or rotation which may affect the shape of the 3D-construct, thus avoiding inaccurately displaying the target lesion. Sudden movements of the patients during the sweep, for example due to discomfort as well as prostate deformation by the application of too much pressure with the probe, can also alter the shape of the 3D-construct [13]. Although fusion platforms attempt to correct these alterations by using elastic registration algorithms and motion compensation, our results suggest the need for further optimization in this field [14]. Of particular importance, a deviation of the needle from the intended and predefined core path, for example due to its asymmetric bevel, is less likely to be compensated in smaller lesion sizes [13]. However, clinicians are partly able to adapt to this veering effect as they become more experienced over time and a (semi-)robotic needle guidance might further reduce the user-dependent effect. In contrast to previously published similar studies, we did not observe a large impact of the urologist’s experience on cancer detection [8–10].

No significant correlation was shown between prostate volume and the occurrence of mismatches, which might be due to the rather small sample size of our cohort. However, inverse association of prostate volume with PCa detection by FB was previously described [15, 16]. It is postulated that an increased prostate volume is associated with the deformation of the prostate during biopsy procedure leading to registration errors. Furthermore, the increased depth of the target lesion as may be found in an enlarged prostate is likely to be associated with increase in deviation of biopsy path [17].

The number of adverse events during and after the procedure are comparable to those in other studies [18].

Interpreting our results, a key limitation is the possibility of different surgical conditions for the first and second surgeon. It is suggested that the accuracy of hitting the target lesion on the real-time TRUS image during the second TB is decreased by tissue swelling caused by the first biopsy procedure. Regarding the discrepancies in csPCa finding, considerations to heterogeneity of tumor lesions should be made. Aihara et al. revealed in PCa specimens that with increased lesion size multiple grades of PCa can be present which are arranged in heterogeneous and unpredictable geographic interrelationships [19]. Therefore, evaluation of each surgeon’s accuracy based on the grade of PCa might be limited. Despite the large number of different urologists taking biopsies, our results are still valuable since they reflect the real-world practice.

This study demonstrates that reproducibility and precision of targeting lesions suspicious for PCa is still limited, even with the high-level support of a semi-robotically software-based fusion biopsy platform. Although the detection rate of PCa and csPCa can be markedly improved by FB, discrepancies in biopsy results between individual urologists can still be observed. This insight should serve as an incentive for further improvements in image fusion, segmentation, needle guidance as well as automatization of the procedure so that even unexperienced clinicians are able to reliably hit a small lesion in an enlarged prostate.

## Supplementary Information

Below is the link to the electronic supplementary material.

Supplementary file1 (TIFF 131 kb) **Online Resource 1** Comparison of patient- and lesion-based detection rates between targeted biopsy 1 (TB1) and targeted biopsy 2 (TB2)

Supplementary file2 (PDF 69 kb) **Online Resource 2** Reliability of PCa detection between target biopsies using Cohen’s kappa coefficient (*κ*)

Supplementary file3 (PDF 108 kb **Online Resource 3** Logistic regression model of factors associated with a mismatch per lesion between both target biopsies

## Data Availability

The raw data supporting the conclusions of this article will be made available by the authors, without undue reservations. Patients signed informed consent regarding publishing their data.
