# Supplementary figures and images for: A prospective study on inter-operator variability in semi-robotic software-based MRI/TRUS-fusion targeted prostate biopsies
Source: World J Urol. 2021 Nov 26;40(2):427–33. doi: 10.1007/s00345-021-03891-3 (PMC8921147; doi:10.1007/s00345-021-03891-3)

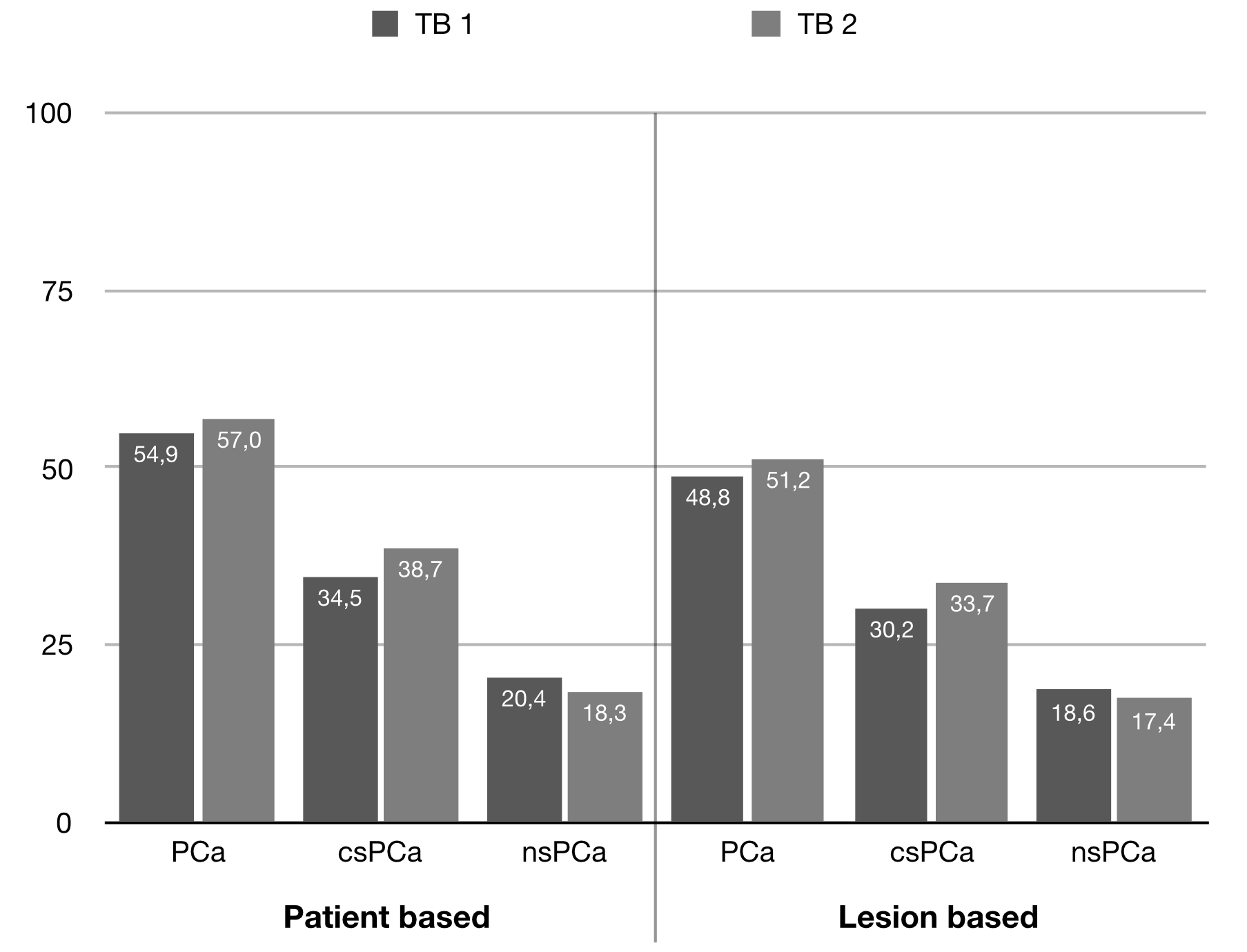

Supplement: Supplementary file 1 — Supplementary file1 (TIFF 131 kb) Online Resource 1 Comparison of patient- and lesion-based detection rates between targeted biopsy 1 (TB1) and targeted biopsy 2 (TB2) [file 345_2021_3891_MOESM1_ESM.tiff]
