# Supplementary material for: A prospective study on inter-operator variability in semi-robotic software-based MRI/TRUS-fusion targeted prostate biopsies
Source: World J Urol. 2021 Nov 26;40(2):427–33. doi: 10.1007/s00345-021-03891-3 (PMC8921147; doi:10.1007/s00345-021-03891-3)
Supplement: Supplementary file 2 — Supplementary file2 (PDF 69 kb) Online Resource 2 Reliability of PCa detection between target biopsies using Cohen’s kappa coefficient (κ) [file 345_2021_3891_MOESM2_ESM.pdf]

Variable	First biopsy – second biopsy<br>*K (95% CI)*
Number of detected PCa	0.56 (0.39 – 0.73)
Number of detected nsPCa	0.56 (0.39 – 0.73)
Number of detected csPCa	0.65 (0.52 – 0.79)
Number of negative TB	0.34 (0.21 – 0.46)

PCa prostate cancer, *csPCa* clinical significant prostate cancer, *nsPCa* insignificant PCa, *SB* systematic biopsy, *TB* targeted biopsy
