# Supplementary material for: A prospective study on inter-operator variability in semi-robotic software-based MRI/TRUS-fusion targeted prostate biopsies
Source: World J Urol. 2021 Nov 26;40(2):427–33. doi: 10.1007/s00345-021-03891-3 (PMC8921147; doi:10.1007/s00345-021-03891-3)
Supplement: Supplementary file 3 — Supplementary file3 (PDF 108 kb Online Resource 3 Logistic regression model of factors associated with a mismatch per lesion between both target biopsies [file 345_2021_3891_MOESM3_ESM.pdf]

Variable	Direction and Unit	Univariable analyses			Multivariable analyses		
		Odds ratio	95% CI	p value	Odds ratio	95% CI	p value
Age <70 (years)	Yes vs. no	0.84	0.31-2.23	0.807			
PSA <7.1 (mg/dl)	Yes vs. no	1.30	0.50-3.42	0.631			
MRI Prostate Volume >41.21 (ml)	Yes vs. no	2.15	0.79-5.84	0.148	1.46	0.47-4.53	0.516
PSA-Density <0.18 (ng/ml^2)	Yes vs. no	1.96	0.74-5.24	0.22	1.57	0.52-4.71	0.4223
DRE	Positive vs. Negative	0.90	0.30-2.72	1.00			
Insignificant PCa	Yes vs. no	2.15	0.78-5.89	0.165	1.52	0.51-4.54	0.458
PI-RADS <4	Yes vs. no	0.78	0.21-2.93	1.00			
Size of the Lesion ≤12 (mm)	Yes vs. no	9.19	2.02-41.83	**<0.001**	8.35	1.81-38.51	**0.007**
Difference in previously performed MRI/TRUS-fusion biopsies between urologist (n)	Increasing units	0.98	0.97-1.00	0.162			
TB cores <3 per urologist (n)	Yes vs. no	0.57	0.19-1.67	0.369			

DRE digital rectal examination, MRI magnetic resonance imaging, PCa prostate cancer, PI-RADS Prostate Imaging Reporting & Data System, PSA prostate-specific antigen, TB targeted biopsy, *TRUS transrectal ultrasound*
